# Controlled Study of Pre‐ and Postoperative Headache in Patients with Sellar Masses (HEADs‐uP Study)

**DOI:** 10.1002/edm2.496

**Published:** 2024-07-12

**Authors:** Tessa N. A. Slagboom, Tessel M. Boertien, Peter H. Bisschop, Eric Fliers, Johannes C. Baaijen, Jantien Hoogmoed, Madeleine L. Drent

**Affiliations:** ^1^ Department of Endocrinology & Metabolism Amsterdam UMC Location Vrije Universiteit Amsterdam Amsterdam The Netherlands; ^2^ Pituitary Centre Amsterdam Amsterdam The Netherlands; ^3^ Amsterdam Gastroenterology Endocrinology and Metabolism Amsterdam The Netherlands; ^4^ Department of Endocrinology & Metabolism Amsterdam UMC Location University of Amsterdam Amsterdam The Netherlands; ^5^ Department of Neurosurgery Amsterdam UMC Location University of Amsterdam Amsterdam The Netherlands; ^6^ Amsterdam Neuroscience Amsterdam The Netherlands

**Keywords:** headache, pituitary tumour, sellar mass, transsphenoidal surgery

## Abstract

**Introduction:**

Sellar masses are common intracranial neoplasms. Their clinical manifestations vary widely and include headache. We aimed to determine whether the prevalence and characteristics of headache in patients with sellar tumours differ from the general population and to investigate the effect of tumour resection on this complaint.

**Methods:**

We performed a prospective, controlled study in a single tertiary centre and included 57 patients that underwent transsphenoidal resection for a sellar mass (53% females, mean age 53.5 ± 16.4) and 29 of their partners (controls; 45% females, mean age 54.8 ± 14.9). Outcome measures were prevalence, characteristics and impact of headache 1 month preoperatively and at neurosurgical follow‐up 3 months postoperatively.

**Results:**

Preoperatively, the prevalence of regular headache (≥1 time per month) was higher in patients than in controls (54% vs. 17%, *p* < 0.001), and patients scored higher on headache impact questionnaires (all *p* ≤ 0.01). At postoperative follow‐up, headache prevalence decreased in both groups, but the decrease in regular headache frequency and impact was larger in patients than in controls, and no between‐group differences remained.

**Conclusions:**

More than half of patients with sellar tumours suffer from at least once‐monthly headaches, and both regular headache occurrence and impact are higher compared with controls. The more pronounced decrease in headache complaints in patients versus controls at postoperative follow‐up suggests an additional effect of tumour resection next to the factor time.

## Graphical abstract

We assessed whether the prevalence and characteristics of headache in patients with sellar tumours differ from the general population and investigated the effect of tumour resection on this complaint. Our study showed that more than half of patients with sellar tumours suffer from at least once‐monthly headaches, and both regular headache occurrence and impact are higher compared with controls. The more pronounced decrease in headache complaints in patients versus controls at postoperative follow‐up suggests an additional effect of tumour resection next to the factor time.

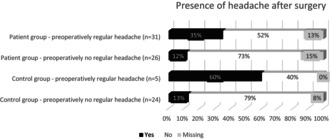

## 1 Introduction

Sellar masses are common intracranial neoplasms with an estimated population prevalence of 0.1% [1, 2]. Pituitary adenomas account for most of these masses. Clinical manifestations of sellar masses vary widely and depend on hormonal status and mechanical symptoms because of tumour expansion. Although many of these masses are asymptomatic, others can lead to endocrine, ophthalmological or neurological complaints, such as headache.

Headache is a frequently encountered (11%–70%), and sometimes predominant, symptom in patients with sellar masses [3, 4]. The pathophysiology behind this common complaint is not fully understood for most sellar pathology, but might be the net result of combined mechanical, biochemical and/or psychological aspects [3, 4, 5, 6, 7]. However, as both headache, with an estimated global prevalence of 52% [8], and (asymptomatic) lesions of the sellar region are common in the general population, it is difficult to establish a causal correlation between the two [9]. The probability that a patient presenting with headache has a concurrent mass in the sellar region as incidental finding on neuroimaging during the diagnostic work‐up (i.e., an ‘incidentaloma’) is considerable [10, 11]. It thus remains unclear to which extent the high prevalence of headache is attributable to effects of the sellar mass and should thereby be classified as secondary headache [12]. Secondary headaches can have the characteristics of a primary headache, that is, a headache without underlying cause or condition [12], further complicating the differentiation between primary and secondary headaches in patients with a sellar tumour if based solely on clinical symptoms. Headache in a patient with a sellar mass, especially if this concerns a hormonal inactive tumour that does not threaten the optic nerves, therefore presents a true management dilemma [13]. At present, not much is known about the characteristics and impact of the headache that these patients experience. Large, prospective studies on the effects of tumour resection on headache in patients with sellar masses are scarce. The available literature to date provides conflicting results regarding the improvement in complaints after transsphenoidal surgery [3, 6, 14, 15, 16].

Headache can have a major impact on the quality of life [9]. Diagnosis and proper treatment of pituitary tumour‐related headache could therefore enhance quality of life to a relevant extent. In order to investigate whether the prevalence and characteristics of headache in patients with sellar tumours differ from the general population and to draw conclusions on the effect of tumour resection versus natural course of this complaint, we performed a prospective, controlled, single‐centre study among patients with sellar masses and their partners, to evaluate the presence, characteristics and impact of headache, pre‐ and postoperatively. Secondly, we investigated determinants of both the presence and impact of, as well as the effect of, tumour resection on headache in patients with sellar masses.

## 2 Materials and Methods

### 2.1 Participants

One hundred consecutive, adult patients with a sellar mass or space‐occupying cystic lesion planned for elective surgical resection at the Amsterdam University Medical Centre (Amsterdam UMC) between June 2019 and January 2022 were invited to participate in the study, after permission of their treating specialist. If present, their partners were invited to participate in the control group. All patients were recruited through the Pituitary Centre Amsterdam, a multidisciplinary outpatient clinic consisting of a neurosurgeon, otolaryngologist and endocrinologist. Exclusion criteria were inflammatory disease affecting the pituitary (e.g., hypophysitis), other major intracranial affliction <3 months preoperatively (e.g., traumatic brain injury, cerebral infarction or intracranial haemorrhage other than pituitary apoplexy), malignant tumours (e.g., pituitary carcinoma and metastases) and inability to give informed consent. The Medical Ethical Committee of the Amsterdam UMC location AMC approved the study protocol.

### 2.2 Study Design

After signing informed consent, participants received baseline questionnaires to assess headache (details below) 1 month in advance of the planned surgery. Participating partners were also asked to fill in a form about their medical history and medication use. Filling in the questionnaires took about 15 min. Three months postoperatively, participants received follow‐up questionnaires. Data on demographics and medical history were gathered using medical files (patients) or through the additional questionnaire/form (partners). Routine clinical practice concerning endocrine status and pituitary imaging according to international guidelines was adhered to in all patients [17, 18, 19]. Preoperative MRI results were used to determine tumour size, extension (including sinus cavernous invasion), compression of optic nerves or chiasm and most probable diagnosis. Prior to surgery, biochemical testing was performed to determine whether the tumour was hormonally active and to assess pituitary deficiencies, with cut‐off scores defined by the Endocrine Society Clinical Practice Guideline [20]. Biochemical testing was performed in the laboratory of the Amsterdam UMC location AMC. All tumour resections were performed via an endoscopic transnasal transsphenoidal approach by a neurosurgeon and otolaryngologist. Three neurosurgeons and four otolaryngologists performed all operations in our centre. Operation details, such as surgical technique and perioperative complications, were documented in an operative report. Resected tumour material underwent pathological and immunohistochemical examination for definitive diagnosis. All patients had a follow‐up visit 3 months postoperatively with an assessment of the following aspects: the presence of residual tumour on a postoperative MRI, resolution of hormonal hypersecretion and/or pituitary deficiencies, resolution of preoperative visual field defects or cranial nerve palsies, new pituitary deficiencies, pathology results and postoperative adjuvant therapies.

### 2.3 Questionnaires

Participants were first asked if they ever experienced headache, and if not, they did not have to fill in the headache questionnaires.

#### 2.3.1 Headache Screening Questionnaire—Dutch Version (HSQ)

This is a validated screening questionnaire for migraine and tension‐type headache (TTH) based on criteria of the Internal Classification of Headache Disorders, 3rd edition [21]. It consists of 10 multiple‐choice questions about the frequency and clinical manifestations of experienced headache. Outcome measures are total migraine and total TTH score. A total score of ≥6 points corresponds with probable migraine or TTH, and a full score of 8 points with definite migraine or TTH. Sensitivity/specificity for detecting probable migraine, definite migraine, probable TTH and definite TTH are 0.89/0.54, 0.69/0.90, 0.92/0.48 and 0.36/0.86, respectively.

#### 2.3.2 General Headache Questionnaire

Further information regarding headache was gathered with a self‐composed questionnaire consisting of 12 items, on the basis of widely used diagnostic tools implemented in headache clinics and designed to complement the Headache Screening Questionnaire. Firstly, the presence of any headache as well as the presence of regular headaches (defined as headache at least once a month) was checked pre‐ and postoperatively. Postoperative headache within 2 weeks after surgery was excluded. If any headache was present, participants were asked to mark the location(s) of their experienced headache on a picture and to check accompanying symptoms that could suggest TACs (e.g., ptosis or rhinorrhoea). Several following questions checked medication use to treat or prevent headache and the effect as well as side effects of used medication. Although we were not able to report on the number of participants with a definite diagnosis of medication‐overuse headache (MOH) based on the International Headache Society (IHS) criteria, we did report on a group that fulfilled two out of three diagnostic criteria for MOH: headache for at least 15 days a month + use of analgesics for at least three times a week. Participants were also asked if they had ever consulted a specialist or other healthcare provider and whether a specific diagnosis or explanation for their headache was given. Additionally, familial occurrence of headache was checked. Finally, the severity of average and worst experienced headache in the last 7 days was quantified using a Visual Analogue Scale (VAS) (100‐mm line without sub marks, from ‘no pain’ to ‘worst imaginable pain’) [22]. The VAS is widely used in diverse clinical settings and has good sensitivity of pain rating [23].

#### 2.3.3 Headache Impact Test (HIT‐6)

This validated questionnaire quantifies the impact of headache on daily life [24, 25]. It consists of six items on how often headache affects daily activities or mood, rated on a 5‐point scale from ‘never’ (6 points) to ‘always’ (13 points). Total score ranges from 36 to 78 points, with higher scores indicating a higher impact on daily life. The HIT‐6 has good discriminative validity and test–retest reliability (Cronbach's α = 0.78) [25].

#### 2.3.4 Headache Severity Index (HSI)

Gfrerer et al. constructed a migraine headache severity index (HSI) score on the basis of headache severity, duration and frequency, which was later also used for NFA by Yu et al. [26, 27]. We calculated our own HSI on the basis of the HIT‐6 score and two questions from the HSQ, with a score ranging from 3 to 12 points (Table 1).

**TABLE 1:** Calculation of the Headache Severity Index (HSI).

Points	*Severity* (HIT‐6 score)	*Duration* (HSQ Question 4)	*Frequency* (HSQ Question 3)
1	≤49	0–30 min	<1 time a month
2	50–55	30 min—4 h	1–15 times a month
3	56–59	4 h—3 days	>15 times a month
4	≥ 60	3–7 days	
5		≥ 7 days	

#### 2.3.5 Tumour‐Related Headache

Schankin et al. proposed three criteria for the definition of tumour‐attributable headache in pituitary adenoma: presence of preoperative headache, exclusion of MOH and ≥50% amelioration of headache postoperatively in respect of frequency or severity [28]. The latter criterion was used for current study, in which we defined ≥50% amelioration of headache as ≥50% reduction in HIS, with the inclusion of patients headache resolution postoperatively.

### 2.4 Statistical Analysis

SPSS Statistics version 28.0.1.1 was used for statistical analysis. Numerical data are summarised as mean ± SD or as median (IQR), depending on distribution. Categorical and ordinal data are presented as counts (%). Between‐group differences in numerical variables were analysed with independent *t*‐tests (normal distributed variables) or the Mann–Whitney *U* (non‐normal distributed variables), and differences in categorical variables with the chi‐squared or Fisher exact (if expected cell count <5) test. Comparisons of pre‐ and postoperative data within the patient group were evaluated by paired *t*‐tests, the Mann–Whitney *U* or chi‐squared test, as appropriate. Correlations were calculated using Pearson correlation coefficient *r* (normal distributed variables) or Spearman's correlation coefficient *ρ* (non‐normal distributed variables). Within the patient group, the following determinants of (presence and impact of) headache were analysed: age, tumour size (in mm), tumour expansion (based on sinus cavernous invasion and/or visual field defects), apoplexy, functioning adenoma (any, and separately for Cushing's disease, acromegaly and prolactinoma), hypopituitarism and complete resection of tumour (based on postoperative MRI results). We also performed subanalyses on pituitary neuroendocrine tumours (PitNET) transcription factors in those patients with a pituitary adenoma (Appendix B). When data were missing or invalid (e.g., in case multiple answers were given), the participant was excluded from the analysis for that variable. Outcomes were considered statistically significant at *p* ≤ 0.05.

## 3 Results

### 3.1 Study Population

**FIGURE 1:**
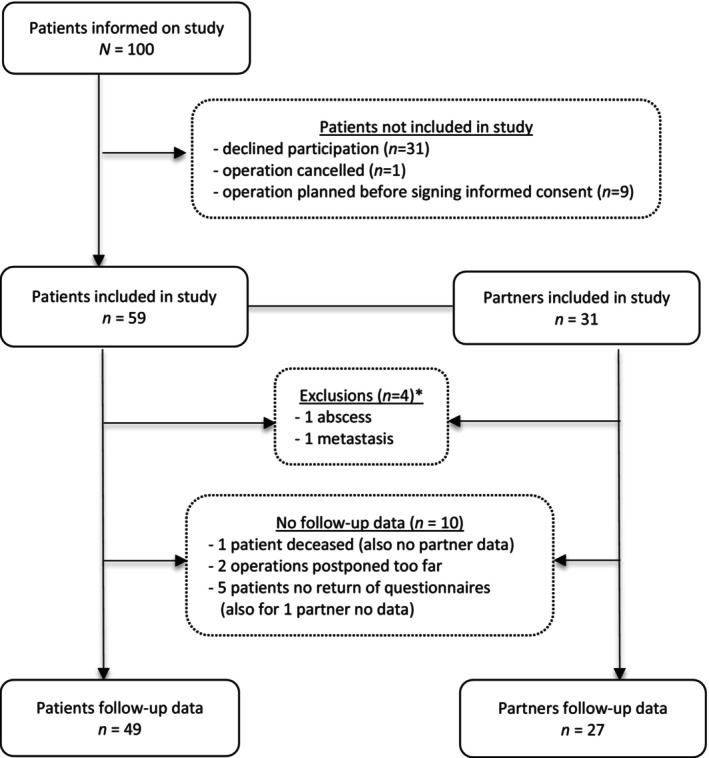
Inclusion flow chart. * of the patients who were excluded because of other diagnosis (*n* = 2; abscess, metastasis), partners (*n* = 2) were also excluded.

Of the 100 patients who received information on the study, 59 (59%) agreed to participate. Of these, 31 (53%) had a partner willing to participate. Two patients and accompanying partners were excluded. Follow‐up data were gathered from 49 patients (83%) and 27 partners (87%). Figure 1 shows the inclusion flow chart with reasons for exclusions and missing follow‐up data. No differences were found between patients with and without follow‐up data with respect to age, gender, adenoma size, percentage of hormonally active tumours and percentage of hypopituitarism (data not shown). Also, no statistical differences in sex or mean age at inclusion were found between patients (53% females, age 53.5 ± 16.4) and controls (45% females, age 54.8 ± 14.9), *p* = 0.49 and 0.72, respectively. Tumour characteristics of patients are summarised in Table 2.

**TABLE 2:** Tumour characteristics and surgical outcomes for the patient group.

Tumour characteristics	Sellar lesion (*n* = 57)	Pituitary adenoma			Total	95%
			Size		In mm (median [IQR])	21.0 [10.0 – 31.8]
					Micro (<1 cm)	22%
					Macro (≥ 1cm)	78%
			Hormonally active	No		65%
				Yes	GH	13%
					ACTH	15%
					TSH	2%
					Prolactin	6%
		Rathke's cleft cyst				4%
		Craniopharyngeoma				2%
	Hypopituitarism (*n* = 57)	No				46%
		Yes			GH	5%
					ACTH	33%
					TSH	32%
					LH/FSH	35%
					ADH	0
	Local tumour effects (*n* = 57)				Chiasm compression	68%
		Visual defect			Any	47%
					Quadrant hemianopsia	4%
					Bitemporal hemianopsia	26%
					Other	18%
					Apoplexy	5%
					Cranial nerve deficit	0
					Sinus cavernous invasion	51%
Surgical outcomes	Time between indication and surgery (*n* = 55^a^)				Average in days [range]	75.2 ± 66.9 [6 – 280]
					% of patients operated <30 days	30%
	Postoperative follow‐up (*n* = 54^b^)				Average (in days)	96.9 ± 39.7
	Postoperative MRI (*n* = 54^b^)				Complete resection of tumour	17%
					Residual tumour	74%
					Uncertain	9%
	Resolution of hormonal hypersecretion (*n* = 19)					58%

	Resolution of preoperative pituitary deficiencies (*n* = 31)				Complete	16%
					Partial	23%
	Visual field defects preoperative (*n* = 27)				Improvement	56%
					Stable	18%
					Requiring further follow‐up	26%
	Adjuvant therapy (*n* = 54^b^)				Additional surgery	17%
					Radiotherapy	4%
				Medication	4%

^a^ Data missing for two patients with postponed surgery.

^b^ Data missing for two patients with postponed surgery and one deceased patient.

### 3.2 Surgery and Follow‐Up

Table 2 shows the surgical outcomes in the patient group. Indications for resection were objectified visual disturbances (46%), hormonal hypersecretion (26%), prevention of visual disturbances (23%) and side effects or contra‐indication for dopamine agonists in case of prolactinoma (5%). No resections were performed with headache as the sole indication. Pathology results confirmed a pituitary adenoma in 48 cases (84%), two Rathke's cleft cysts and one craniopharyngioma. For pituitary adenoma, the clinicopathological classification can be found in Appendix B.

### 3.3 Between‐Group Comparisons

#### 3.3.1 Occurrence

**FIGURE 2:**
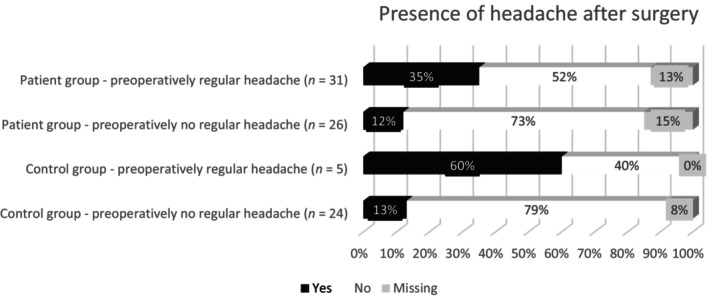
The presence of pre‐ and postoperative headache in patients and controls.

Preoperatively, a similar proportion of patients (47/57 [82%]) and controls (23/29 [79%]) had ever experienced headache (χ^2^(1, *N* = 86) = 0.13, *p* = 0.72). Postoperatively, headache was reported in 14/49 (29%) patients and 6/27 (22%) controls (χ^2^(1, *N* = 76) = 0.36, *p* = 0.55) (Table 3). Before surgical treatment, regular headache (≥1 time per month) was present more often in patients than in controls (54% vs. 17%, χ^2^(1, *N* = 86) = 10.9, *p* < 0.001), whereas this difference was not found after surgery (27% vs. 15%, χ^2^(1, *N* = 76) = 1.38, *p* = 0.55). No new cases of headache were reported in either group, but headache frequency worsened from <1 time a month to 1–15 times a month for two patients and two controls. Of the participants with missing follow‐up data, seven out of the eight patients reported to ever having experienced headache preoperatively (of which three regular headaches), and none of the two controls. Figure 2 shows the course of headache in four groups of participants, based on the presence of regular (≥1 per month) preoperative headache (yes/no) and group (patient/controls). McNemar test in patients showed that the chance of regular headache resolution after transsphenoidal resection (16/27) was greater than the chance of experiencing headache postoperatively in patients who did not report regular headache preoperatively (3/22—of which 2 worsened from <1 a month to 1–15 times a month and one remained the same with frequency < 1 per month), *p* = 0.004; versus *p* = 1.00 in controls.

**TABLE 3:** Headache variables.

			Patients				Controls			
			Preoperatively		Postoperatively		Preoperatively		Postoperatively	
Does headache occur in your family? (Yes)				25/57 (44%)		N/A		11/29 (38%)		N/A
Have you ever experienced a headache? (Yes)				47/57 (82%)		N/A		23/29 (79%)		N/A
Did you experience a headache, ≥2 weeks after surgery? (Yes)				N/A		14/49 (29%)		N/A		6/27 (22%)
Do you experience regular headache (≥1 per month)? (Yes)				31/57 (54%)*^&^		13/49 (27%) ^&^		5/29 (17%)*		4/27 (15%)
How often in your life have you had a headache?	1–4 times		*n =* 47	6%	*n* = 14	0%	*n* = 23	9%	*n* = 6	0%
	5–9 times			9%		14%		13%		0%
	≥ 10 times			85%		86%		78%		100%
How often per month do you experience a headache?	<1 time		*n* = 47	34%	*n* = 14	7%	*n* = 23	78%	*n* = 6	33%
	1 to 15 times			43%		86%		22%		67%
	≥ 15 times			23%*		7%		0%*		0%
How often did you have a headache attack?	0–4 times		*n* = 46	41%	*n* = 14	57%	*n* = 23	52%	*n* = 6	50%
	5–9 times			17%		14%		17%		0%
	≥ 10 times			41%		29%		30%		50%
How long does your headache last, without medication?	0–30 min		*n* = 45	22%	*n* = 14	14%	*n* = 23	13%	*n* = 6	0%
	30 min. – 4 h			42%		64%		70%		67%
	4 h—3 days			24%		21%		17%		33%
	3–7 days			4%		0%		0%		0%
	>7 days			7%		0%		0%		0%
Is your headache one‐ or two‐sided	One‐sided		*n* = 37	46%	*n* = 14	50%	*n* = 21	57%	*n* = 6	83%
	Two‐sided			54%		50%		43%		17%
Which word would you use to describe your headache	Pulsing		*n* = 43	12%	*n* = 12	0%	*n* = 22	14%	*n* = 6	0%
	Tight or pressing			58%		83%		68%		83%
	Burning or stinging			16%		17%		5%		17%
	Other			14%		0%		14%		0%
Headache accompanying symptoms	Sensitivity to light		*n* = 47	40%	*n* = 14	36%	*n* = 23	48%	*n* = 6	50%
	Sensitivity to noise			19%*		21%		48%*		50%
	Nausea/vomiting			17%		14%		17%		0%
	Other			38%**		7%		13%**		17%
	Cranial autonomic symptoms	Facial transpiration		11%		14%		22%		0%
		Swollen eyelid		11%		0%		0%		0%
		Ptosis		11%		14%		13%		17%
		Conjunctival hyp. and lacr.		21%		7%		9%		33%
		Myosis		2%		0%		4%		0%
		Rhinorrhoea and nasal cong.		19%**		21%		0%**		0%
Describe the seriousness of your headache	Mild		*n* = 44	30%	*n* = 14	29%	*n* = 23	30%	*n* = 6	0%
	Moderate			39%		57%		52%		83%
	Serious			25%		7%		9%		17%
	Very serious			7%		7%		9%		0%
Do you use any medication to stop your headache?	No		*n* = 44	34%	*n* = 14	36%	*n* = 22	32%	*n* = 6	17%
	Per attack			11%		7%		14%		50%
	<1 time a month			16%		0%		28%		0%
	1–8 times a month			11%		21%		18%		33%
	3–5 times a week			11%		21%		5%		0%
	Daily			18%		21%		5%		0%
Total score on the HSQ	Possible migraine		*n* = 36	19%	*n* = 13	31%	*n* = 22	32%	*n* = 6	33%
	Migraine			5%		8%		9%		17%
	Possible tension‐type headache		*n* = 38	82%	*n* = 12	75%	*n* = 22	73%	*n* = 6	100%
	Tension‐type headache			37%		42%		50%		67%
	Both possible migraine + tension‐type headache		*n* = 36	17%	*n* = 12	14%	*n* = 22	23%	*n* = 6	33%

*Note*: Comparisons between groups were conducted, and if significant indicated by *p ≤ 0.01, **p ≤ 0.05; within groups by ^&^ *p* ≤ 0.01.

Abbreviations: con., congestion; conjunctival hyp. and lacr., conjunctival hyperaemia and lacrimation; *n*, number of responders per item.

#### 3.3.2 Characteristics

**FIGURE 3:**
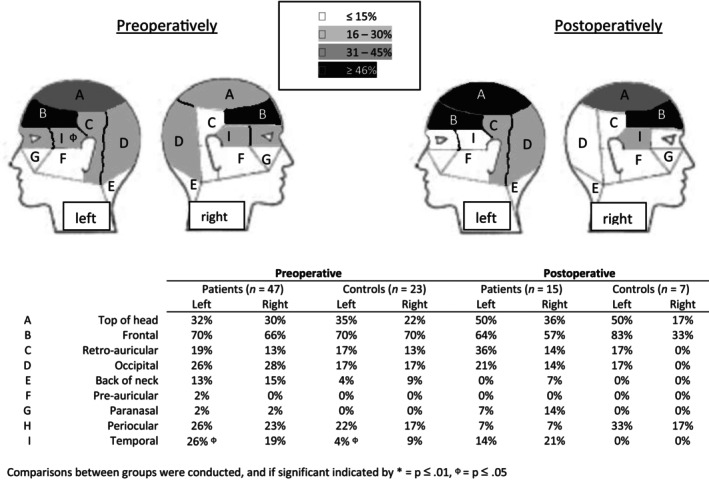
Pre‐ and postoperative location of headache in patients and controls. Multiple headache locations were possible per participant.

Frontal headache was reported most frequently in both groups, and besides a higher frequency of left‐sided temporal pain for patients preoperatively (*p* = 0.05), no differences between the two groups were found (Figure 3). Preoperatively, controls experienced significantly more sensitivity to noise (*p* = 0.01), whereas patients experienced more rhinorrhoea and nasal congestion (*p* = 0.03), and other symptoms (*p* = 0.03) including fatigue, dizziness, pressure and loss of concentration. Symptoms suggestive of a form of trigeminal autonomic cephalalgias (TAC) (i.e., one‐sided headache with a usual duration less than 30 min) were present in five patients before surgery, of which three also in combination with accompanying symptoms of facial transpiration, swollen eyelid or conjunctival hyperaemia and lacrimation, versus no controls. These headaches where predominantly frontal (3/5) and occipital (2/5) and did not meet the HSQ criteria for migraine or tension‐type headache (three possible tension‐type headache and one possible migraine), whereas one patient previously received a diagnosis of migraine and another of cluster headache. HIT scores were 0 in two patients (no or little impact on daily life), 1 in two patients (some impact on daily life) and 3 in one patient (considerable impact on daily life). None of these patients used medication to stop their headaches. The diagnoses included one patient with acromegaly (microadenoma without expansion), one patient with Cushing's disease (microadenoma without expansion) and three nonfunctional pituitary adenomas (macroadenoma with parasellar and sinus cavernous extension, also one with suprasellar expansion). Postoperatively, headache resolution occurred in 3/5 patients, persisted in 1/5 (with same HIT score: 3) and was unknown in one patient because the surgery was postponed.

Because of missing or double/ambiguous answers on the HSQ questions (especially the question about one‐ or two‐sided headache), total score for (possible) migraine or TTH could not be calculated for, respectively, 11 and 9 patients, and 7 controls. Preoperatively, six patients (13%) fulfilled two of three MOH criteria versus none of the controls (*p* = 0.17), whereas no participants fulfilled two of three MOH criteria postoperatively.

#### 3.3.3 Impact

Regarding headache impact and severity, patients had statistically significant higher average scores on the HIT‐6, VAS and HSI preoperatively, indicating more severe headache with higher impact on daily life (Table 4). Although postoperative differences in HIT‐6 and VAS still seem apparent, these differences could not be demonstrated in a statically significant manner, possibly due to top low group numbers. Tumour‐related headache, defined as an improvement of HSI of ≥50% (including participants with headache resolution postoperatively) was found in 76% of both patients as controls.

**TABLE 4:** Impact scores.

		Patients				Controls			
		Preoperatively		Postoperatively		Preoperatively		Postoperatively	
Daily activities worsen my headache (Yes)		*n* = 45	24%	*n* = 14	29%	*n* = 23	17%	*n* = 6	33%
I avoid daily activities during my headache (Yes)		*n* = 46	22%	*n* = 14	21%	*n* = 23	43%	*n* = 6	33%
HIT‐6	Total	52.2 ± 9.8*		53.6 ± 12.1		46.1 ± 6.5*		48.5 ± 6.0	
	<49 (no or little effect on daily life)	*n* = 46	41%	*n* = 14	50%	*n* = 25	68%	*n* = 6	67%
	50–55 (some effect on daily life)		22%		14%		24%		17%
	56–59 (considerable effect on daily life)		7%		7%		4%		17%
	≥ 60 (severe effect on daily life)		31%**		29%		4%**		0%
VAS	Average headache score past week	20.2 [4.5–58.3]*		22.6 [9.7–36.6]		1.5 [0.0–7.5]*		3.5 [1.5–24.4]	
	Worst headache score past week	20.9 [4.0–67.0]*		30.0 [16.8–55.4]		1.5 [0.0–15.9]*		3.9 [1.8–30.9]	
HSI	Total	8.0 [3.0–18.0]**		6.0 [4.0–18.0]		2.0 [2.0–4.0]**		4.0 [3.5–9.8]	
	Improvement in HSI score ≥ 50%			*n* = 37	76%			*n =* 16	76%

*Note: n*, number of responders per item/participants of which a sum of score could be calculated. Comparisons between groups were conducted and, if significant, indicated by *p ≤ 0.01, **p ≤ 0.05.

#### 3.3.4 Diagnosis Other than Pituitary Adenoma

We performed sensitivity analyses excluding patients with a diagnosis other than pituitary adenoma (Rathke's cleft cyst [*n* = 2] and craniopharyngeoma [*n* = 1]), but this did not significantly change the results (data not shown).

##### 3.3.4.1 Within Patient Comparisons

Although the prevalence of regular headache significantly lowered after surgery in the patient group (Table 4), the impact of headache (measured by mean HIT‐6, median average and worst VAS and median HSI score) did not significantly change from preoperative status to postoperative follow‐up (all *p* > 0.05). Preoperatively, the only significant headache determinants were age on presence of headache (*p* = 0.01) and presence of hormonal hypersecretion on headache impact (*p* = 0.02) (Appendix A). A greater impact of headache postoperatively was found in patients with hormonal hypersecretion (*p* = 0.006, especially GH secretion *p* = 0.03) and lack of tumour expansion (no sinus cavernous invasion: *p* = 0.008, no preoperative visual defect: *p* = 0.008). A strong negative association between postoperative headache impact and tumour size was found (*ρ* = −0.88). Within patients with a pituitary adenoma, the influence of transcription factors (T‐PIT, SF‐1 and PIT‐1) and Ki‐67 index on presence and impact of pre‐ and postoperative headache was determined (Appendix B). The absence of SF‐1 (*p* = 0.002) and presence of PIT‐1 (*p* = 0.006) were determinants of headache impact postoperatively.

## 4 Discussion

In this prospective study, we found that although the preoperative prevalence of any headache was similar, patients with a sellar mass suffered from more frequent and more intense headaches with a higher impact on daily life when compared to controls. Furthermore, these differences were nonsignificant at postoperative follow‐up, possibly indicating a positive effect from transsphenoidal surgery on severity and impact of headache in these patients. Since the presence of headache lowered in both groups, we suggest that the factor of time also plays a role in resolution of this complaint. Finally, we identified multiple determinants (age, hormonal hypersecretion, tumour size and expansion, and transcription factors) of both presence and impact of headache in patients with sellar masses.

Preoperative prevalence of regular monthly headache in patients with sellar masses was 54% in the current study, which corroborates with previously reported prevalence rates ranging between 37% and 70% [15, 16, 27, 28, 29, 30, 31, 32]. Concerning headache characteristics, we found that most patients experienced a tension‐type‐like headache (61%), followed by undefined headache (21%), mixed tension‐type‐ and migraine‐like headache (16%) and migraine‐like headache (3%). This is contradictive to other studies finding a higher prevalence of migraine‐like than tension‐type‐like headaches in these patients [15, 27, 33]. With respect to headache location, we found more temporally located headache in patients; however, this is nondistinctive for a specific headache diagnosis. Although no official diagnosis of TACs could be established by our questionnaires, five patients reported short (<30 min), unilateral headache attacks preoperatively, with 3/5 patients also experiencing accompanying symptoms of facial transpiration, swollen eyelid or conjunctival hyperaemia with lacrimation; this combination was not found in any of the controls and postoperative headache resolution occurred in three of them. Some previous studies have described cases of TACs in relatively small sample sizes of patients with pituitary tumours, indicating a higher prevalence of these headaches than what could be expected from numbers in the general population [15, 28, 33, 34], but this might also be due to selection bias (e.g., more often actively asked upon). Next to a higher monthly prevalence of preoperative headache, impact and severity of headache were also higher in patients than controls. Our data show that headache had a severe effect on daily life in 31% of patients versus in 4% of controls. This is comparable to previous studies using the Migraine Disability Assessment (MIDAS) questionnaire as instrumental tool for headache disability in pituitary tumours, with headache affecting daily life severely in 21% to 48% of patients [15, 16, 33]. We found a higher impact of preoperative headache in patients with hormonal hypersecretion (HIT‐6). This is in line with previous studies showing the highest impact (disability) of headache in acromegaly and prolactinoma [6, 16, 33]. Of the 31/57 patients who were experiencing regular headache before surgery, more than half did not report this at postoperative follow‐up. This is in concurrence with previous studies demonstrating resolution of headache in 40%–63% of patients [14, 15, 16, 27, 35]. However, these studies did not include a control group to account for the factor of time in resolution of complaints. We found that the decrease in regular headache prevalence at follow‐up was much more prominent in the patient group than in the control group, lowering the between‐group difference to nonsignificance. This clearly suggests a positive effect of transsphenoidal surgery additional to the factor time.

As in other studies, no predictors on the resolution of headache could be identified [3, 14]. Concerning headache severity based on the HSI, we found improvement in 76%, worsening in 11% and equal scores in 11% of patients (data partly shown), which is in line with previous studies (improvement 49%–83%; worsening 6%–17%, equal 12%–36%) [3, 14, 16, 27, 28, 32, 33]. In contrast to three other studies, we found no new cases of headache after surgery [14, 15, 16]. Two of these studies were prospective and showed 10/34 (30%) and 4/85 (5%) new headache cases, the other was retrospective and identified 5/56 (9%) new cases. The discrepancy between current and previously mentioned studies could be due to timing of follow‐up: on average 3 months in our study versus 6 months or more in the other studies. Contrary to Jahangiri et al., we found no effect on complete resection versus residual tumour on headache presence or impact postoperatively [3].

Various determinants of headache impact in current study share a common factor: functional adenoma. These adenomas were more often diagnosed at a younger age, microadenomas without tumour expansion and SF‐1 negative/PIT‐1 positive (data not shown). Together, this suggests that the presence, severity and impact of headache depend on biochemical factors more than on mechanical factors. PIT‐1 positive tumours and GH overproduction alone were associated with more impact of headache during postoperative follow‐up. Headache is a commonly described complaint in both acromegaly and prolactinoma, and while aetiology is not yet unravelled, it is speculated that these hormones inhibit nociceptive peptide(s) [13]. Medical therapy aimed at reducing hormonal hypersecretion generally has positive effect on headaches, although for somatostatin analogues that result might also be due to antinociceptive effects rather than normalisation of hormonal levels. Moreover, (severe) headache exacerbations have been reported in dopamine agonists [33, 36, 37, 38, 39, 40, 41]. To clarify a possible relationship between tumour size and headache frequency and impact in nonfunctioning sellar masses, we performed sensitivity analyses excluding secretory adenomas, but found no changes (no relationship between tumour size and headache occurrence, negative relationship between tumour size and postoperative headache impact, data not shown). A different hypothesis on headache and sellar masses, is that tumour volume and extension or invasion in nearby structures play a less important role than an increase in intrasellar pressure, which can especially be present in microadenomas without destruction of surrounding bone tissue [5, 42, 43]. Hayashi et al. evaluated headache impact according to the HIT‐6 and intrasellar pressure during transsphenoidal surgery in 108 patients with pituitary adenoma and hypothesised that headache as a result of increased intrasellar pressure was due to dura mater stretch in the sella turcica [43]. However, a study by Pereira‐Neto et al. in 25 pituitary adenoma patients failed to show a relationship between intrasellar pressure and headache impact, also based on HIT‐6 scores. The fact that in our population impact of headache after surgery negatively correlated with tumour size also argues against this hypothesis, since one would expect intrasellar pressure to normalise after transsphenoidal removal of the tumour.

Besides mechanical and biochemical aspects, psychosocial factors also seem to play an important role in the pathophysiology, experiencing and suffering of headache in patients with sellar tumours [4]. Two recent studies by Siegel et al. found that in a group of 112 patients with sellar masses, just like in primary headache, the presence of and disability due to headache were associated with depression, neuroticism and pain catastrophising [7, 15]. However, our study did not assess psychological factors.

A major strength of the current study is the prospective assessment of headache; an important feature in patient reported outcomes to minimise recall bias. Furthermore, by including a control group consisting of patients' partners with similar sociodemographic and ‐economic status, we minimised environmental bias. Unfortunately, not all patients provided a participating partner. We are also the first to include the new PitNET classification in relation to headache in our pituitary adenoma patients. One limitation of the current study is that not all participants fully completed the questionnaire, or gave ambiguous (e.g., multiple) answers. In order to lose as little data as possible, we have chosen to exclude missing data per variable. Since we only included patients that were scheduled for elective transsphenoidal surgery, a selection bias was raised, excluding patients with sellar masses requiring no surgery (e.g., small NFA or medically treated prolactinoma) or emergency surgery (e.g., apoplexy involving fast visual deterioration). Also, the COVID‐19 pandemic changed usual practice temporarily, with expedited surgery for patients with an absolute indication (e.g., clear visual field defects). This shortened the time window for inclusion in the study, whereas surgery was postponed for patients with smaller (nonfunctioning) tumours. Besides, it is difficult to distinguish a new headache due to the sellar mass in patients, as the diagnostic delay of sellar masses is usually long or because of previous therapy such as transsphenoidal surgery, thereby lacking timeline information [44]. Another limitation is that participants were not individually interviewed in order to gain more detailed information. Since, therefore, the exact time course of the complaints is unknown, we could not diagnose ‘headache attributed to intracranial neoplasm’ or TACs according to the ICHD‐3 [12]. Lastly, the duration of follow‐up was quite short in this study. Although a longer follow‐up is desirable to better understand the further course of headache, the 3 month period was chosen to coincide with the regular postoperative follow‐up in these patients.

To our best knowledge, this is the first prospective study on headache in patients with sellar tumours that included a control group. Our results contribute to improvement of management of this complaint, by showing the effect of surgical treatment and determining predictors of headache occurrence and impact. In order to manage expectations and to ameliorate quality of life in these patients, we suggest that more attention to and consultation on headache is given.

In conclusion, we demonstrate that about half of patients with sellar tumours suffer from monthly, mostly tension‐type‐like, headache and that both regular headache occurrence and impact are higher compared with controls. At postoperative follow‐up, the decrease in regular headache frequency and impact was more pronounced in patients than in controls. We also found that the impact of headache was dependent on secretory status, with no clear association with mechanical factors. In order to further evaluate whether the chance of headache improvement should be taken into account during counselling for transsphenoidal surgery of a sellar mass, we suggest future research to explore this complaint in larger studies that include structured patient interviews, and possibly add an additional group consisting of patients with sellar masses without an operation indication to provide more data on specific headache diagnoses and the natural course of these complaints.

## Author Contributions

**Tessa N. A. Slagboom:** conceptualization (lead), data curation (lead), formal analysis (lead), investigation (lead), methodology (equal), project administration (equal), writing–original draft (lead). **Tessel M. Boertien:** conceptualization (lead), investigation (lead), methodology (equal), project administration (equal), supervision (lead), writing–original draft (lead), writing–review and editing (equal). **Peter H. Bisschop:** conceptualization (equal), writing–review and editing (equal). **Eric Fliers:** conceptualization (equal), writing–review and editing (equal). **Johannes C. Baaijen:** conceptualization (equal), writing–review and editing (equal). **Jantien Hoogmoed:** conceptualization (equal), writing–review and editing (equal). **Madeleine L. Drent:** conceptualization (lead), supervision (lead), writing–review and editing (lead).

## Conflicts of Interest

The authors declare no conflicts of interest.

## Data Availability

The datasets generated during and/or analyzed during the current study are available from the corresponding author on reasonable request.

## Appendix A Subanalyses on the influence of multiple determinants on presence and impact of headache and tinnitus, before and after operation within the patient group.

### A.1

**Table:**

	Headache			
	Presence of headache^a^		Impact (HIT‐6 score)	
	Preoperatively	Postoperatively	Preoperatively	Postoperatively
Age (in years)				
With	46.9 ± 15.1	50.7 ± 16.4	*ρ* = −0.29	*ρ* = −0.25
Without	58.7 ± 15.5	53.1 ± 16.4		
*p*	0.01*	0.64		
Tumour size (in mm)				
With	20.0 ± 12.2	24.1 ± 13.8	*ρ* = −0.26	*ρ* = −0.88*
Without	25.0 ± 15.0	22.1 ± 14.0		
*p*	0.20	0.65		
Sex				
Male	12/27 (44%)	7/23 (30%)	50.7 ± 8.7	55.8 ± 15.4
Female	19/30 (63%)	7/26 (27%)	53.5 ± 10.7	52.0 ± 10.3
*p*	0.15	0.79	0.35	0.62
Sinus cavernous invasion				
Yes	16/29 (55%)	8/25 (32%)	51.1 ± 10.0	45.2 ± 8.8
No	15/28 (54%)	6/24 (25%)	53.5 ± 9.8	62.0 ± 8.7
*p*	0.90	0.59	0.40	0.008*
Visual defect preoperative				
Yes	15/27 (56%)	6/24 (25%)	51.6 ± 10.2	43.6 ± 7.1
No	16/30 (53%)	8/25 (32%)	52.8 ± 9.8	60.7 ± 9.7
*p*	0.87	0.59	0.70	0.008*
Apoplexy				
Yes	2/3 (67%)	2/3 (67%)	53.0 ± 15.6	53.5 ± 10.6
No	29/54 (54%)	12/46 (26%)	52.3 ± 9.8	53.6 ± 13.0
*p*	1.00	0.19	0.92	0.99
Complete resection				
Yes	4/9 (44%)	2/2 (100%)	50.9 ± 10.4	59.0 ± 7.1
No	27/46 (59%)	11/12 (92%)	53.5 ± 9.4	52.5 ± 12.9
*p*	0.47	1.00	0.52	0.39
Hormonal hypersecretion				
Yes	12/19 (63%)	4/15 (27%)	56.8 ± 9.4	65.8 ± 8.4
No	19/38 (50%)	10/34 (29%)	49.8 ± 9.3	47.5 ± 8.6
*p*	0.35	1.00	0.02**	0.006*
Adrenocorticotrophic hormone producing				
Yes	5/9 (56%)	2/7 (29%)	53.9 ± 9.4	60.5 ± 2.1
No	24/45 (53%)	12/39 (31%)	52.4 ± 10.1	52.2 ± 12.9
*p*	1.00	1.00	0.72	0.40
Growth hormone producing				
Yes	4/7 (57%)	1/6 (17%)	60.0 ± 11.2	78.0 (*n* = 1)
No	25/47 (53%)	13/40 (33%)	51.7 ± 9.4	51.4 ± 9.9
*p*	1.00	0.65	0.08	0.03**
Prolactinoma				
Yes	3/3 (100%)	1/2 (50%)	59.0 ± 6.1	64.0 (*n* = 1)
No	26/51 (51%)	13/44 (30%)	52.2 ± 10.0	52.6 ± 12.3
*p*	0.24	0.52	0.18	0.40
Hypopituitarism				
Yes	19/31 (61%)	8/27 (30%)	50.44 ± 10.35	51.3 ± 13.4
No	12/26 (46%)	6/22 (27%)	54.74 ± 8.87	56.8 ± 10.7
*p*	0.25	0.86	0.16	0.46

^a^ preoperatively defined as regular headache (≥1 a month), postoperatively defined as headache >2 weeks surgery; Pre‐ and postoperative comparisons within patients were conducted and, if significant, indicated by **p* ≤ 0.01; ***p* ≤ 0.05.

## Appendix B Pathology outcomes for *n* = 54 pituitary adenoma.

### B.1

**Table:**

Transcription factor		Immunophenotypes		Tumour type	
T‐PIT +	9 (17%)	ACTH +	8 (15%)	Corticotroph adenoma	9 (17%)
SF‐1 +	28 (52%)	FSH +	24 (44%)	Gonadotroph adenoma	28 (52%)
		LH +	20 (37%)		
		ɑ‐subunit +	12 (22%)		
PIT‐1 +	11 (20%)	GH +	6 (11%)	Somatotroph adenoma	2 (4%)
		PRL +	7 (13%)	Lactotroph adenoma	4 (7%)
		ɑ‐subunit +	4 (7%)	Mammosomatotroph adenoma	4 (7%)
		TSH +	1 (2%)	Plurihormonal (GH, PRL, TSH, ɑ‐subunit)	1 (2%)
Unknown	6 (11%)	Reasons: ‐ surgery postponed (2 nonfunctioning adenoma) ‐ too little material for pathology (1 acromegaly, 1 Cushing's disease) ‐ normal pituitary tissue (1 Cushing's disease) or variable pathology outcome (1 Cushing's disease)			

	Headache				
	Presence of regular headache			Impact (HIT‐6 score)	
	Preoperatively		Postoperatively	Preoperatively	Postoperatively
SF‐1	Yes	13/28 (46%)	8/26 (31%)	50.3 ± 9.4	44.0 ± 6.5
	No	13/20 (65%)	5/16 (31%)	55.1 ± 9.5	63.4 ± 8.8
	*p*	0.20	1.00	0.14	0.002*
PIT‐1	Yes	7/11 (64%)	3/9 (33%)	57.6 ± 10.0	67.7 ± 9.1
	No	19/37 (51%)	10/33 (30%)	50.9 ± 9.1	47.3 ± 8.2
	*p*	0.47	1.00	0.07	0.006*
T‐PIT	Yes	6/9 (67%)	2/7 (29%)	52.4 ± 8.7	57.0 ± 2.8
	No	20/39 (51%)	11/35 (31%)	52.6 ± 10.0	51.9 ± 13.7
	*p*	0.48	1.00	0.96	0.63
Ki‐67	≤1%	7/19 (37%)	6/18 (33%)	52.4 ± 10.6	58.0 ± 16.7
	2%	17/27 (63%)	5/22 (23%)	52.1 ± 9.6	49.0 ± 10.8
	≥ 3%	2/2 (100%)	2/2 (100%)	62.0 ± 2.8	60.0 ± 1.4
	*p*	0.09	0.13	0.59	0.48

*Note:* Pre‐ and postoperative comparisons within patients were conducted and, if significant, indicated by **p* ≤ 0.01, ***p* ≤ 0.05.

## References

[1] K. Al‐Dahmani , S. Mohammad , F. Imran , et al., “Sellar Masses: An Epidemiological Study,” Canadian Journal of Neurological Sciences 43, no. 2 (2016): 291–297. 10.1017/cjn.2015.301 26522017

[2] T. A. Dolecek , J. M. Propp , N. E. Stroup , and C. Kruchko , “CBTRUS Statistical Report: Primary Brain and Central Nervous System Tumors Diagnosed in the United States in 2005–2009,” Neuro‐Oncology 14, no. suppl_5 (2012): v1–v49. 23095881 10.1093/neuonc/nos218 PMC3480240

[3] A. Jahangiri , J. R. Wagner , A. T. Chin , et al., “Incidence of Headache as a Presenting Complaint in Over 1000 Patients With Sellar Lesions and Factors Predicting Postoperative Improvement,” Clinical Neurology and Neurosurgery 132 (2015): 16–20. 25746316 10.1016/j.clineuro.2015.02.006

[4] I. Kreitschmann‐Andermahr , S. Siegel , R. Weber Carneiro , J. M. Maubach , B. Harbeck , and G. Brabant , “Headache and Pituitary Disease: A Systematic Review,” Clinical Endocrinology 79, no. 6 (2013): 760–769. 23941570 10.1111/cen.12314

[5] B. M. Arafah , D. Prunty , J. Ybarra , M. L. Hlavin , and W. R. Selman , “The Dominant Role of Increased Intrasellar Pressure in the Pathogenesis of Hypopituitarism, Hyperprolactinemia, and Headaches in Patients With Pituitary Adenomas,” The Journal of Clinical Endocrinology & Metabolism 85, no. 5 (2000): 1789–1793. 10843153 10.1210/jcem.85.5.6611

[6] A. Wolf , S. Goncalves , F. Salehi , et al., “Quantitative Evaluation of Headache Severity Before and After Endoscopic Transsphenoidal Surgery for Pituitary Adenoma,” Journal of Neurosurgery 124, no. 6 (2016): 1627–1633. 26495954 10.3171/2015.5.JNS1576

[7] S. Siegel , T. Schenk , G. Brabant , R. C. Scholl , M. Buchfelder , and I. Kreitschmann‐Andermahr , “Not Simply a Structural Problem: Psychological Determinants of Headache in Patients With Tumors of the Sellar Region,” Experimental and Clinical Endocrinology & Diabetes 130, no. 10 (2022): 693–700. 35977692 10.1055/a-1851-5017

[8] L. J. Stovner , K. Hagen , M. Linde , and T. J. Steiner , “The Global Prevalence of Headache: An Update, With Analysis of the Influences of Methodological Factors on Prevalence Estimates,” The Journal of Headache and Pain 23, no. 1 (2022): 34. 35410119 10.1186/s10194-022-01402-2 PMC9004186

[9] L. Stovner , K. Hagen , R. Jensen , et al., “The Global Burden of Headache: A Documentation of Headache Prevalence and Disability Worldwide,” Cephalalgia 27, no. 3 (2007): 193–210. 17381554 10.1111/j.1468-2982.2007.01288.x

[10] S. Ezzat , S. L. Asa , W. T. Couldwell , et al., “The Prevalence of Pituitary Adenomas: A Systematic Review,” Cancer: Interdisciplinary International Journal of the American Cancer Society 101, no. 3 (2004): 613–619. 10.1002/cncr.20412 15274075

[11] M. L. Mahesar , A. Jatoi , N. A. Shaikh , A. K. Narsani , and M. L. Mahesar , “Frequency of Pituitary Adenoma Among the Patients With Chronic Headache,” Journal of Pharmaceutical Research International 33, no. 60B (2021): 1235–1241.

[12] Headache Classification Committee of the International Headache Society (IHS) , “The International Classification of Headache Disorders, (Beta Version),” Cephalalgia 33, no. 9 (2013): 629–808. 23771276 10.1177/0333102413485658

[13] M. J. Levy , M. Matharu , and P. J. Goadsby , “Chronic Headache and Pituitary Tumors,” Current Pain and Headache Reports 12, no. 1 (2008): 74–78. 18417028 10.1007/s11916-008-0014-5

[14] G. B. Gravdahl , E. A. Tronvik , S. L. Fougner , and O. Solheim , “Pituitary Adenoma and Non‐acute Headache: Is there an Association, and Does Treatment Help?” World Neurosurgery 92 (2016): 284–291. 27132176 10.1016/j.wneu.2016.04.071

[15] S. Siegel , R. Weber Carneiro , M. Buchfelder , et al., “Presence of Headache and Headache Types in Patients With Tumors of the Sellar Region—Can Surgery Solve the Problem? Results of a Prospective Single Center Study,” Endocrine 56, no. 2 (2017): 325–335. 28243973 10.1007/s12020-017-1266-9

[16] A. Andersson , T. Hallén , D. S. Olsson , et al., “Headache before and after Endoscopic Transsphenoidal Pituitary Tumor Surgery: A Prospective Study,” Journal of neurological surgery. Part B, Skull Base 83, no. S 02 (2021): e360–e366. 35832989 10.1055/s-0041-1729180 PMC9272269

[17] L. K. Nieman , B. M. Biller , J. W. Findling , et al., “Treatment of Cushing's Syndrome: An Endocrine Society Clinical Practice Guideline,” The Journal of Clinical Endocrinology and Metabolism 100, no. 8 (2015): 2807–2831. 26222757 10.1210/jc.2015-1818 PMC4525003

[18] L. Katznelson , E. R. Laws Jr , S. Melmed , et al., “Acromegaly: An Endocrine Society Clinical Practice Guideline,” The Journal of Clinical Endocrinology and Metabolism 99, no. 11 (2014): 3933–3951. 25356808 10.1210/jc.2014-2700

[19] P. U. Freda , A. M. Beckers , L. Katznelson , et al., “Pituitary Incidentaloma: An Endocrine Society Clinical Practice Guideline,” The Journal of Clinical Endocrinology & Metabolism 96, no. 4 (2011): 894–904. 21474686 10.1210/jc.2010-1048 PMC5393422

[20] M. Fleseriu , I. A. Hashim , N. Karavitaki , et al., “Hormonal Replacement in Hypopituitarism in Adults: An Endocrine Society Clinical Practice Guideline,” The Journal of Clinical Endocrinology & Metabolism 101, no. 11 (2016): 3888–3921. 27736313 10.1210/jc.2016-2118

[21] H. A. van der Meer , C. M. Visscher , R. H. H. Engelbert , W. M. Mulleners , M. W. G. Nijhuis – van der Sanden , and C. M. Speksnijder , “Development and Psychometric Validation of the Headache Screening Questionnaire–Dutch Version,” Musculoskeletal Science and Practice 31 (2017): 52–61. 28734169 10.1016/j.msksp.2017.07.001

[22] W. Van der Kloot , R. Oostendorp , J. Van der Meij , and J. Van Den Heuvel , “The Dutch Version of the McGill Pain Questionnaire: A Reliable Pain Questionnaire,” Nederlands Tijdschrift voor Geneeskunde 139, no. 13 (1995): 669–673. 7723868

[23] M. J. Hjermstad , P. M. Fayers , D. F. Haugen , et al., “Studies Comparing Numerical Rating Scales, Verbal Rating Scales, and Visual Analogue Scales for Assessment of Pain Intensity in Adults: A Systematic Literature Review,” Journal of Pain and Symptom Management 41, no. 6 (2011): 1073–1093. 21621130 10.1016/j.jpainsymman.2010.08.016

[24] M. Martin , B. Blaisdell , J. W. Kwong , and J. B. Bjorner , “The Short‐Form Headache Impact Test (HIT‐6) Was Psychometrically Equivalent in Nine Languages,” Journal of Clinical Epidemiology 57, no. 12 (2004): 1271–1278. 15617953 10.1016/j.jclinepi.2004.05.004

[25] M. Kosinski , M. S. Bayliss , J. B. Bjorner , et al., “A Six‐Item Short‐Form Survey for Measuring Headache Impact: The HIT‐6™,” Quality of Life Research 12, no. 8 (2003): 963–974. 14651415 10.1023/a:1026119331193

[26] L. Gfrerer , D. Y. Maman , O. Tessler , and W. G. J. Austen , “Nonendoscopic Deactivation of Nerve Triggers in Migraine Headache Patients: Surgical Technique and Outcomes,” Plastic and Reconstructive Surgery 134, no. 4 (2014): 771–778. 24945947 10.1097/PRS.0000000000000507

[27] B. Yu , N. Ji , Y. Ma , B. Yang , P. Kang , and F. Luo , “Clinical Characteristics and Risk Factors for Headache Associated With Non‐functioning Pituitary Adenomas,” Cephalalgia 37, no. 4 (2017): 348–355. 27154998 10.1177/0333102416648347

[28] C. J. Schankin , A. K. Reifferscheid , M. Krumbholz , et al., “Headache in Patients With Pituitary Adenoma: Clinical and Paraclinical Findings,” Cephalalgia 32, no. 16 (2012): 1198–1207. 23059488 10.1177/0333102412462639

[29] M. J. Levy , H. R. Jäger , M. Powell , M. S. Matharu , K. Meeran , and P. J. Goadsby , “Pituitary Volume and Headache: Size Is Not Everything,” Archives of Neurology 61, no. 5 (2004): 721–725. 15148150 10.1001/archneur.61.5.721

[30] A. Pereira‐Neto , A. M. Borba , P. A. Mello , L. A. Naves , A. S. Araújo Jr , and L. A. Casulari , “Mean Intrasellar Pressure, Visual Field, Headache Intensity and Quality of Life of Patients With Pituitary Adenoma,” Arquivos de Neuro‐Psiquiatria 68 (2010): 350–354. 20602034 10.1590/s0004-282x2010000300004

[31] J. A. Gondim , J. P. C. de Almeida , L. A. F. de Albuquerque , M. Schops , É. Gomes , and T. Ferraz , “Headache Associated With Pituitary Tumors,” The Journal of Headache and Pain 10, no. 1 (2009): 15–20. 19067118 10.1007/s10194-008-0084-0 PMC3451766

[32] T. Abe , K. Matsumoto , J. Kuwazawa , I. Toyoda , and K. Sasaki , “Headache Associated With Pituitary Adenomas,” Headache: The Journal of Head and Face 38, no. 10 (1998): 782–786. 10.1046/j.1526-4610.1998.3810782.x 11279904

[33] M. Levy , M. S. Matharu , K. Meeran , M. Powell , and P. J. Goadsby , “The Clinical Characteristics of Headache in Patients With Pituitary Tumours,” Brain: A Journal of Neurology 128, no. 8 (2005): 1921–1930. 15888539 10.1093/brain/awh525

[34] P. Chitsantikul and W. J. Becker , “SUNCT, SUNA and Pituitary Tumors: Clinical Characteristics and Treatment,” Cephalalgia 33, no. 3 (2013): 160–170. 23197348 10.1177/0333102412468672

[35] E. Valassi , B. M. Biller , A. Klibanski , and B. Swearingen , “Clinical Features of Nonpituitary Sellar Lesions in a Large Surgical Series,” Clinical Endocrinology 73, no. 6 (2010): 798–807. 20874772 10.1111/j.1365-2265.2010.03881.x PMC2982869

[36] M. J. Levy , P. Bejon , M. Barakat , P. J. Goadsby , and K. Meeran , “Acromegaly: A Unique Human Headache Model,” Headache 43, no. 7 (2003): 794–797. 12890136 10.1046/j.1526-4610.2003.03139.x

[37] N. R. Musolino , R. Marino , and M. D. Bronstein , “Headache in Acromegaly: Dramatic Improvement With the Somatostatin Analogue SMS 201–995,” The Clinical Journal of Pain 6, no. 3 (1990): 243–245. 2135020 10.1097/00002508-199009000-00013

[38] I. Schreiber , M. Buchfelder , M. Droste , et al., “Treatment of Acromegaly With the GH Receptor Antagonist Pegvisomant in Clinical Practice: Safety and Efficacy Evaluation from the German Pegvisomant Observational Study,” European Journal of Endocrinology 156, no. 1 (2007): 75–82. 17218728 10.1530/eje.1.02312

[39] M. J. Levy , “The Association of Pituitary Tumors and Headache,” Current Neurology and Neuroscience Reports 11, no. 2 (2011): 164–170. 21128024 10.1007/s11910-010-0166-7

[40] M. Gabrielli , A. Gasbarrini , G. Fiore , et al., “Resolution of Migraine With Aura after Successful Treatment of A Pituitary Microadenoma,” Cephalalgia 22, no. 2 (2002): 149–150. 11972585 10.1046/j.1468-2982.2002.00314.x

[41] M.‐M. Kallestrup , H. Kasch , T. Østerby , E. Nielsen , T. S. Jensen , and J. O. L. Jørgensen , “Prolactinoma‐Associated Headache and Dopamine Agonist Treatment,” Cephalalgia 34, no. 7 (2014): 493–502. 24351278 10.1177/0333102413515343

[42] Y. Hayashi , D. Kita , M. Iwato , et al., “Significant Improvement of Intractable Headache after Transsphenoidal Surgery in Patients With Pituitary Adenomas; Preoperative Neuroradiological Evaluation and Intraoperative Intrasellar Pressure Measurement,” Pituitary 19, no. 2 (2016): 175–182. 26659379 10.1007/s11102-015-0696-8

[43] Y. Hayashi , Y. Sasagawa , M. Oishi , et al., “Contribution of Intrasellar Pressure Elevation to Headache Manifestation in Pituitary Adenoma Evaluated With Intraoperative Pressure Measurement,” Neurosurgery 84, no. 3 (2019): 599–606. 29618106 10.1093/neuros/nyy087

[44] T. Brue and F. Castinetti , “The Risks of Overlooking the Diagnosis of Secreting Pituitary Adenomas,” Orphanet Journal of Rare Diseases 11, no. 1 (2016): 135. 27716353 10.1186/s13023-016-0516-x PMC5052978

